# Establishment of two new human bladder carcinoma cell lines, CAL 29 and CAL 185. Comparative study of cell scattering and epithelial to mesenchyme transition induced by growth factors

**DOI:** 10.1054/bjoc.2001.2105

**Published:** 2001-09-01

**Authors:** N Cattan, N Rochet, C Mazeau, E Zanghellini, B Mari, C Chauzy, H Stora de Novion, J Amiel, J-L Lagrange, B Rossi, J Gioanni

**Affiliations:** Laboratoire de Cancérologie, Centre Antoine Lacassagne, Av. Valombrose, Nice, 06189; INSERM Unité 364; Faculté de Médecine, INSERM Unité 526, Av. Valombrose, Nice, 06107; Laboratoire d'Oncologie Moléculaire, Centre Henri Becquerel, Rouen, 76000; Laboratoire Génazur, 73 Bd Henri Sappia, Nice, 06100; Service d'Urologie, Centre Hospitalo-Universitaire, 30 voie Romaine, Nice, 06000; Service de Radiothérapie, Centre Antoine Lacassagne, Av. Valombrose, Nice, 06189, France

**Keywords:** bladder carcinoma, cell scattering, EMT, EFGR ligands

## Abstract

We describe here two new human urothelial carcinoma cell lines, CAL 29 and CAL 185, established from two patients with high-grade tumours and which display very different properties in vitro. We have shown that CAL 29 cells were tumorigenic in mice and expressed characteristic features of both cell scattering and transition from epithelial to mesenchymal phenotype (EMT) after triggering by the EGF receptor ligands, TGFα and EGF. At the opposite, the CAL 185 cells were not tumorigenic in mice and neither scattered nor expressed vimentin intermediary filaments in the presence of growth factors. We further demonstrated that CAL 29 cell scattering was reversible after growth factor removal and that both scattering and EMT were markedly impaired after treatment with MEK, Src and PI3-kinase inhibitors suggesting that these kinases might be important components of the cellular responses to EGF and TGF-α leading to scattering and EMT. These agents could help to understand the intracellular pathways involved in invasiveness and to find new targets for limiting metastasis. In conclusion, these two new cell lines could be good models to dissect the molecular mechanisms involved in invasion and metastasis development in human bladder cancer. © 2001 Cancer Research Campaign  http://www.bjcancer.com


*British Journal of Cancer* (2001) **85**(9), 1412–1417
© 2001 Cancer Research Campaign
doi: 10.1054/ bjoc.2001.2105, available online at http://www.idealibrary.com on IDEAL

http://www.bjcancer.com

### Establishment of two new human bladder carcinoma cell lines, CAL 29 and CAL 185. Comparative study of cell scattering and epithelial to mesenchyme transition induced by growth factors

**N Cattan[1], N Rochet[2], C Mazeau[1], E Zanghellini[1], B Mari[3], C Chauzy[4], H Stora de Novion[5], J Amiel[6], J-L Lagrange[7], B Rossi[2] and J Gioanni[1]**

[1]Laboratoire de Cancérologie, Centre Antoine Lacassagne, Av. Valombrose, 06189 Nice; [2]INSERM Unité 364; [3]INSERM Unité 526, Faculté de Médecine, Av. Valombrose, 06107 Nice; [4]Laboratoire d'Oncologie Moléculaire, Centre Henri Becquerel, 76000 Rouen; [5]Laboratoire Génazur, 73 Bd Henri Sappia, 06100 Nice; [6]Service d'Urologie, Centre Hospitalo-Universitaire, 30 voie Romaine, 06000 Nice; and [7]Service de Radiothérapie, Centre Antoine Lacassagne, Av. Valombrose, 06189 Nice, France

**Summary** We describe here two new human urothelial carcinoma cell lines, CAL 29 and CAL 185, established from two patients with high-grade tumours and which display very different properties in vitro. We have shown that CAL 29 cells were tumorigenic in mice and expressed characteristic features of both cell scattering and transition from epithelial to mesenchymal phenotype (EMT) after triggering by the EGF receptor ligands, TGFα and EGF. At the opposite, the CAL 185 cells were not tumorigenic in mice and neither scattered nor expressed vimentin intermediary filaments in the presence of growth factors. We further demonstrated that CAL 29 cell scattering was reversible after growth factor removal and that both scattering and EMT were markedly impaired after treatment with MEK, Src and PI3-kinase inhibitors suggesting that these kinases might be important components of the cellular responses to EGF and TGF-α leading to scattering and EMT. These agents could help to understand the intracellular pathways involved in invasiveness and to find new targets for limiting metastasis. In conclusion, these two new cell lines could be good models to dissect the molecular mechanisms involved in invasion and metastasis development in human bladder cancer. © 2001 Cancer Research Campaign http://www.bjcancer.com

**Keywords**: bladder carcinoma; cell scattering; EMT; EFGR ligands

Bladder cancer is one of the most commonly diagnosed malignancies in Western societies and its incidence and prevalence are still rising. Eighty percent of these tumours are superficial transitional cell carcinomas (TCC) but exhibit a high recurrence rate (50–80%). Intense research has been conducted to better understand the progression of these superficial tumours to invasive disease. Many studies have been devoted in defining the role of epidermal growth factor receptor (EGFR) ligands in carcinomas and the over-expression of EGFR has been strongly correlated with the transition from superficial to invasive bladder cancer (Imai et al, 1995; Ravery et al, 1995; Thiery and Chopin, 1999). Moreover, the levels of two specific EGFR ligands, namely EGF and TGFα have been shown to be significantly higher in malignant tissues than in normal bladder samples, either spontaneously or after iatrogenic urothelial trauma (Cooper and See, 1992; Mellon et al, 1996). These autocrine loops could thus participate to the growth stimulation of these tumours (Ruck and Paulie, 1998). In the NBT-II rat bladder carcinoma cell line, these growth factors (GF) have been shown to induce a cell scattering and a reversible epithelial-mesenchymal transition (EMT) (Boyer et al, 1989a; 1989b; Thiery and Chopin, 1999) suggesting that they can behave as scatter factors and induce tumour cell dispersion.

*Received 18 January 2001*
*Revised 17 July 2001*
*Accepted 7 August 2001*

*Correspondence to:* N Rochet

Conversion from an epithelial to a mesenchymal phenotype occurs during normal embryo development at several critical stages where cell migration and extra-cellular matrix (ECM) invasion are required. This normal process, temporally and spatially restricted, is highly controlled (Hay, 1995). It is now demonstrated that this transition is also clearly relevant to the mechanisms governing the metastatic progression of carcinomas and, in this case, it has been correlated with a poor outcome of the disease (Birchmeier et al, 1996; Gilles et al, 1996). In vitro, EMT results in cell motility, loss of epithelial morphology and acquisition of mesenchymal characteristics such as expression of vimentin intermediate filaments. It can be induced either by altering the functional integrity of adhesion complexes (Behrens et al, 1989), by expression of matrix metalloproteinases (Lochter et al, 1997) or by activation of various tyrosine kinase receptors (Hay, 1995).

To date EMT studies in urothelial carcinomas has been essentially carried out in animal models and very few information are available on EMT mechanisms in human bladder carcinoma. Here, we report the characterization of two human bladder cell lines CAL 29 and CAL 185, established from patients with high-grade tumours. We have shown that these cell lines differ significantly in morphology, tumorigenicity and biologic behaviour, especially cell scattering and EMT induced by growth factors like TGF-α, EGF, HGF and aFGF. We propose these two cell lines as interesting models to investigate the molecular mechanisms involved in tumour progression towards invasiveness.

### MATERIALS AND METHODS

### Patients and cell culture

The CAL 29 cell line was established from an 80-year-old woman with a grade-IV stage-T2 invasive transitional cell carcinoma (TCC) of the bladder. At the time of diagnosis, she had developed osseous metastases. Despite radiotherapy, the patient died 2 months after resection of the tumour. The CAL 185 was established from a 66-year-old man presented with a grade-III stage-T3 invasive TCC of the bladder. At the time of diagnosis, no metastases were detected. Ten months after local resection and treatment with chemotherapeutic agents (ifosfamide, vimplastin, cisplatinum), local recurrences occurred and the patient died 8 months later with pulmonary and cerebral metastases.

The same protocol was used for the establishment of CAL 29 and CAL 185 cell lines. Briefly, from the primary lesion, explants of 1–2 mm diameter were placed on the bottom of a screw-top culture flask (Falcon, Los Angeles, CA), covered with a small amount of complete nutritive medium to allow adherence of the explants to the plastic. The permanent cell lines CAL 29 and CAL 185 were obtained respectively 6 and 14 months after continuous culture corresponding to 40 passages. Cells were then maintained in DMEM with Earle's salts (Boehringer-Mannheim, Meylan, France) supplemented with 2 mmol/L-glutamine, 10% of fetal calf serum, 400 U/ml penicillin and 200 mg/ml streptomycin. Cells were cultured at 37˚C in a 5% $CO_2$ atmosphere and frozen in liquid nitrogen at different passages.

### Doubling time

$50 \times 10^3$ CAL 29 cells at the 18th passage and $28 \times 10^3$ CAL 185 cells at the 26th passage, were plated in 25 $cm^2$ plastic flasks containing complete medium. Twenty-four hours after plating and every day for 10 days cells were harvested with a solution of 0.05% trypsin in PBS and counted.

### Tumorigenicity

Six-week-old athymic nude mice with the Swiss genetic background (IFFA Credo, 1' Arbresle, France) were used. $10^7$ CAL 29 cells and 7.5 $10^6$ CAL 185 cells were inoculated S.C. in 0.2 ml Ringer's lactate solution in both flanks of two mice. Tumours were inspected at the sites of injection twice a week for 5 months.

### Chromosomal analysis

Cytogenetic studies of the two cell lines were performed at the 20th passage. For each cell line, cells in the exponential phase of growth were treated with colchicine (Sigma, Saint Quentin Fallavier, France) for 1 h at a final concentration of 10 µg/ml. Cells were then trypsinized, washed and treated with a hypotonic solution of tri-sodium citrate (0.3 M). They were then fixed in acetic acid: methanol (1:3, v:v) and placed onto grease-free, cooled slides for chromosomal examination. R bands were obtained by heat denaturation of the chromosomes in Earle's solution (Dutrillaux and Lejeune, 1971).

### Immunofluorescence studies

Cells were cultured on 8 wells chamber slides (Polylabo, Strasbourg, France) in complete medium with 0.5% fetal calf serum, until 20–30% confluence. The immunofluorescence studies were performed at various times before and after treatment by growth factors. When indicated the cells were pre-treated for 2 h with the PI3-kinase inhibitor LY 294002 at 20 µM (Calbiochem, La Jolla, CA), the MEK inhibitor PD 98059 at 30 µM (Calbiochem) or the Src-like kinase inhibitor PP2 at 30 µM (Calbiochem) before growth factor addition. Slides were fixed with acetone at –20˚C for 10 min, then incubated with primary mouse monoclonal antibodies directed either against vimentin at 1/100 dilution (DAKO, Trappes, France), or cytokeratin at 1/50 (KL1, Immunotech, Marseille, France), or GC 12 raised in our laboratory (Gioanni et al, 1993) for 60 min. After washing, anti-mouse FITC-conjugated antibodies (DAKO) were then added for 30 min. Nuclei were stained with propidium iodide after fixation in ethanol for 5 min. Vimentin positive cells were counted by two different investigators.

### Cell scattering assay

Cells were seeded at 20–30% confluence in 8 wells-chamber slides (Polylabo) in DMEM with 0.5% SVF. Recombinant human growth factors were then added at the following concentrations: HGF 50 ng/ml (R&D Systems, Abingdon, UK), EGF 10 ng/ml (Genzyme, Cambridge, MA), TGFα 50 ng/ml and aFGF 50 ng/ml (Boehringer-Mannheim). For morphological analysis, the coverslips were placed on a glass slide in mounting medium, and pictures were taken immediately using the $10 \times$ or $20 \times$ objective on an Olympus C2 microscope. When indicated, kinase inhibitors were added in the culture medium as described above.

### RESULTS

### Morphology by light microscopy

As shown in Figure 1A, CAL 29 cells grew with anchorage dependence and displayed a polyhedric shape with low refringent aspect. Figure 1B shows that CAL 185 cells were polyhedric, refringent and smaller than CAL 29 cells. Nuclear abnormalities were often observed, with prominent nucleoli. Cells grew in compact bundles characteristic of the loss of anchorage dependence.

### Doubling time, tumorigenicity and chromosomal analysis

The population doubling time measured during the period of exponential growth was 30 h at passage 18 for CAL 29 cells and 72 h at passage 26 for CAL 185 cells (not shown). Chromosomal analysis of CAL 29 and CAL 185 did not reveal particular features (Figure 2A and Figure 2B). The chromosome number was 69 for CAL 29 (hyperdiploid) and more heterogeneous for CAL 185 (between 62 and 68). No chromosome markers were found for these two cell lines.

From two series of experiments, we concluded that only CAL 29 cells were tumorigenic in nude mice. Indeed, 2 months after injection, small tumours of 3-mm diameter appeared in two different sites. As shown in Figure 3, histological examination of the xenograft concluded to a malpighian carcinoma, with a moderate differentiation and surrounded by a slight stromal reactive tissue. CAL 185 cells did not induce tumours in nude mice since no tumour was detected 6 months after injection.

© 2001 Cancer Research Campaign

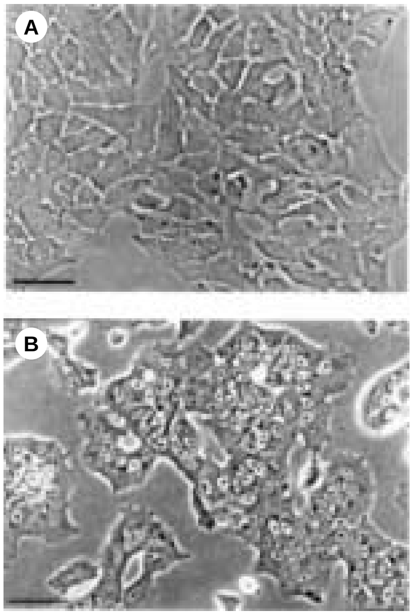

**Figure 1** (**A**) Polyhedric aspect of cell line CAL 29 with anchorage dependence growth. Scale bar 100 µm. (**B**) CAL 185 cells. Small refringent epithelial cells with prominent nucleoli. Cells show anchorage independence growth. Scale bar: 100 µm

### Analysis of epithelial markers and epithelial junction molecules

The reactivity of anti-cytokeratin antibodies, specific for cells of the epithelial cell lineage, was strong and intracytoplasmic for both CAL 29 and CAL 185. In contrast, no reactivity of the GC12 MAb against its mesenchymal antigen was seen in either cell line (data not shown). These results confirm the epithelial origin of CAL 29 and CAL 185 cells.

### Cell scattering triggered by growth factors

Several lines of evidence have indicated that growth factors can behave as tumour cell scattering factors when added to subconfluence cell cultures. We have compared the ability of TGFα, EGF, HGF and aFGF to promote colony dispersion in CAL 29 and CAL 185 cells. After 8 h, morphological analysis demonstrated that these factors were able to induce the dispersion of tightly packed CAL 29 colonies into motile, single cells with spindle-like fibroblastoid appearance. All the growth factors tested were equivalent in their ability to promote cell scattering in CAL 29 cells. It is noteworthy that this scattering was reversible by removal of the growth factor. The results obtained with TGFα are illustrated in Figure 4. At the opposite, CAL 185 cells did not scatter after treatment with any of these factors.

### TGFα and EGF only are able to induce EMT in the CAL 29 cell line

Besides cell scattering, we asked whether all these growth factors were also able to induce EMT. In this aim the two cell lines were exposed to TGFα, EGF, HGF, aFGF as described in Material and Methods and then analysed by immunofluorescence experiments for the expression of the mesenchyme specific intermediate filament, vimentin. In the absence of GF, no vimentin-positive cells could be observed in both cell lines. As represented in Figure 5, in the presence of EGF (Figure 5B) or TGFα (Figure 5C) a small but significant proportion of CAL 29 cells became vimentin positive.

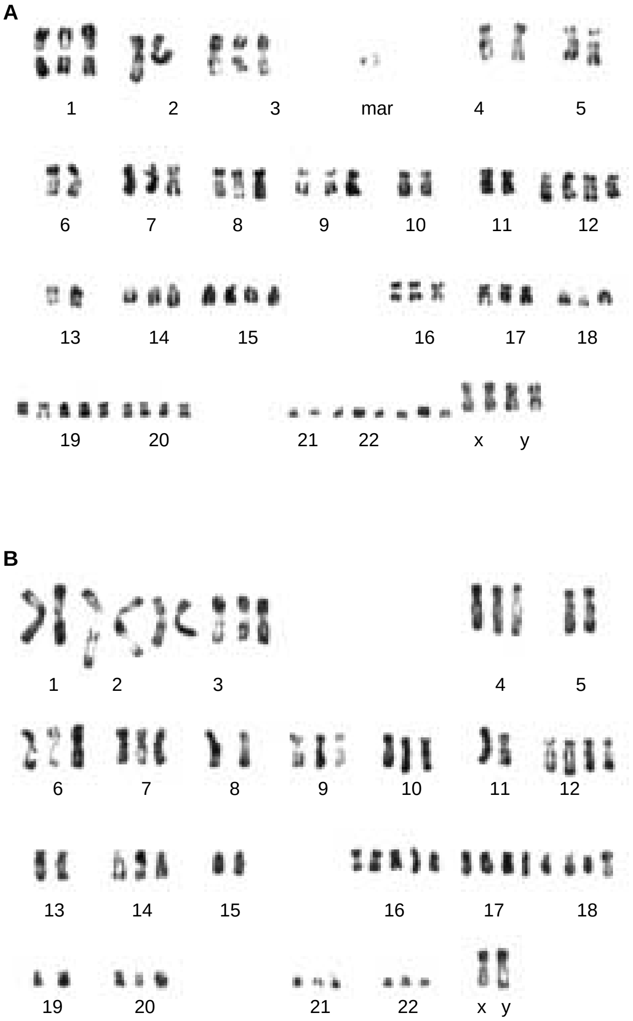

**Figure 2** R-banding karyotype of (**A**) CAL 29 metaphase and (**B**) CAL 185 metaphase

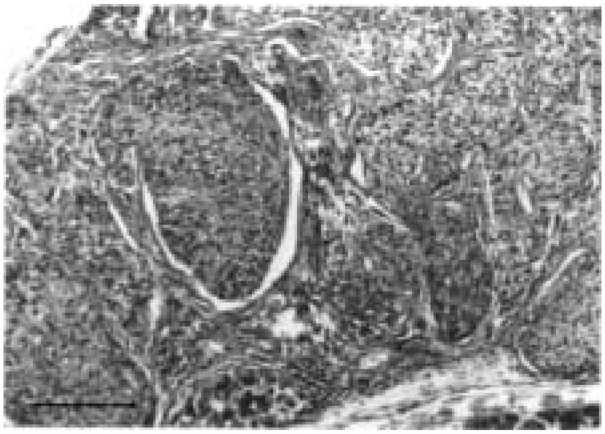

**Figure 3** Histological analysis of one section from a CAL 29 xenografted tumour showing a moderately differentiated squamous cell carcinoma. Scale bar: 100 µm

 © 2001 Cancer Research Campaign

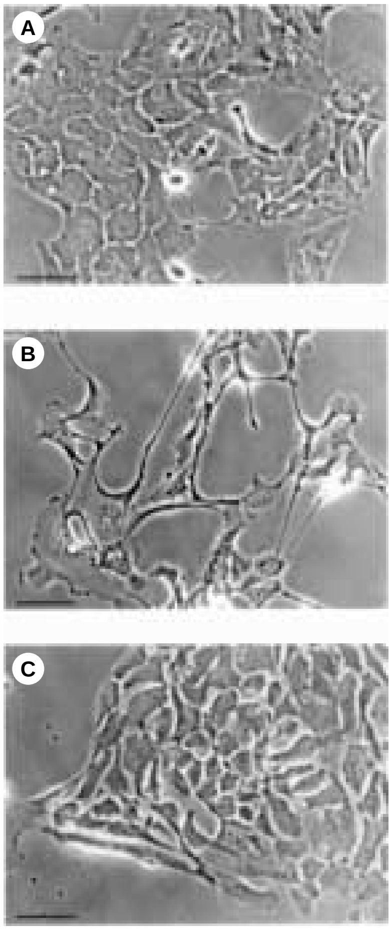

**Figure 4** Scattering effect of TGFα on CAL 29 cells (**A**) before treatment, (**B**) 48 h after treatment, (**C**) reversion of scattering 48 h after TGFα removal. Scale bar 100 μm

This effect was not observed in the presence of HGF and aFGF even after 48 h (Figure 5D and Figure 6A). Vimentin expression was maximal with TGFα after 24-h treatment (5.5%) and decreased to reach about 1% after 72 h (Figure 6B), this percentage was maintained during 6 days. We have observed that EMT was largely reduced after withdrawal of EGF and TGFα but a few cells remained vimentin-positive even 3 days after EGFR ligand deprivation (data not shown). Unlike CAL 29 cells, the CAL 185 cells exhibited no vimentin labelling after GF treatment (data not shown). These data clearly indicated that among these GF, the two EGFR ligands only, namely TGFα and EGF, were able to induce both cell scattering and EMT. Moreover they show that, at the opposite of cell scattering, EMT is not completely reversible after GF deprivation.

### Inhibition of cell scattering and EMT induced by TGFα

Various protein kinases have been involved in growth factor signalling leading to epithelial cell scattering (Boyer et al, 1997; Khwaja et al, 1998). In order to investigate whether Src, MAP kinase and PI3-kinase participate to cell scattering and EMT process, CAL 29 cells were treated with the PI3-kinase inhibitor LY294002, the MEK inhibitor PD98059, the Src-like kinase inhibitor PP2 for 2 h prior to the addition of TGFα. The cell dispersion and the percentage of vimentin positive cells were estimated after 2 days. We have observed that the three inhibitors were able to strongly inhibit TGFα-induced dispersion of CAL 29 cells (data not shown). As shown in Figure 7 for TGFα, these agents were also able to prevent EMT: indeed in the presence of PD98059, LY294002 or PP2, the percentage of vimentin-positive cells was decreased to 14%, 60% and 23% respectively, compared to the basal level obtained in the presence of TGFα alone.

### DISCUSSION

Cell scattering and epithelial-mesenchymal transition (EMT) have been described as hallmarks of tumour progression, metastatic properties and poor outcome of cancer. To date, EMT mechanisms in urothelial carcinomas have been mainly studied in the NBT-II rat bladder carcinoma cells (Thiery and Chopin, 1999). In the present study, we have analysed the role of several growth factors (GF) on EMT induction in two new human urothelial carcinoma cell lines, CAL 29 and CAL 185. These two permanent cell lines express typical characteristics of epithelial cells such as a cytokeratin positive and vimentin negative labelling and the presence of desmosomes and adherens junction proteins (data not shown). Both cell lines have an hyperdiploid karyotype without specific chromosome markers. Cross-contamination with the T24 and 647V human urothelial cell lines was excluded (data not shown). CAL 29 cells, but not CAL 185, were tumorigenic in nude mice, and xenografted tumours exhibited histopathologic features reminiscent of the malpighian structure of the original tumour. We have shown that CAL 29 cells were able to dissociate in response to several growth factors, including EGF, TGFα, HGF and aFGF as assessed by the rupture of desmosomal structures and other cell-cell adhesion complexes observed after immunofluorescent labelling (data not shown). GF-induced spreading was reversible and could be strongly inhibited by MEK, Src and PI-3 kinase inhibitors, indicating that early transduction pathways involved in cell spreading seem to be common to these growth factors.

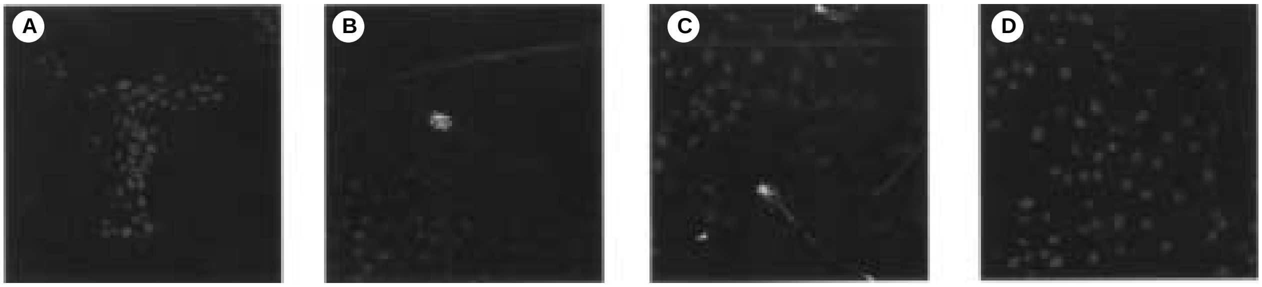

**Figure 5** Vimentin expression by CAL 29 cells (**A**) untreated cells, (**B**) EGF treated cells. (**C**) TGF α treated cells, (**D**) HGF treated cells

© 2001 Cancer Research Campaign

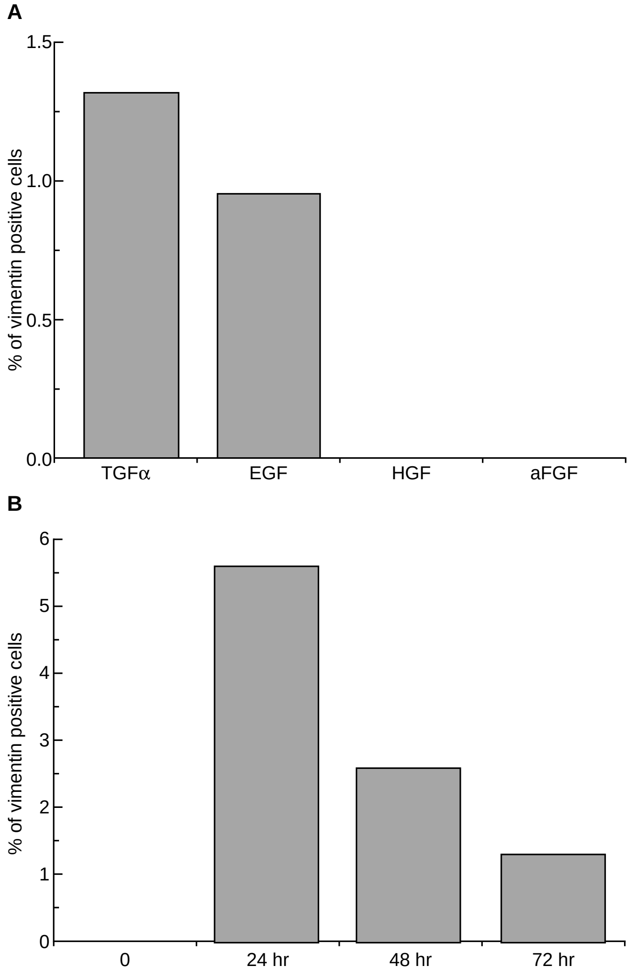

**Figure 6** Vimentin expression by CAL 29 cells. The number of vimentin-positive cells was evaluated after immunofluorescence labelling and counting (**A**) after treatment for 48 h with TGFα, EGF, HGF or aFGF (**B**) Time-course of vimentin induction under TGFα

In spite of the presence of functional receptors for HGF and aFGF, as confirmed by the strong effect of both ligands on cell scattering and by RT-PCR (not shown), HGF and aFGF were not able to trigger EMT. Indeed, vimentin-expressing cells were only detectable in the presence of EGFR ligands, EGF and TGFα, which have been already described as autocrine growth factors for bladder cancer cell lines (Gravilovic et al, 1990; Ruck and Paulie, 1998). These results reinforce the importance of the observation showing that iatrogenic urothelial trauma can induce a marked increase of urinary levels of TGFα (Cooper and See, 1992). Moreover, we have shown that withdrawal of TGFα and EGF did not lead to a complete reversion of EMT, even after 4 days, as described by Lochter and co-workers for mammary cells exposed to stromelysin-1 (Lochter at al, 1997). These results are slightly different from those obtained by Thiery and co-workers which have shown a reversible transition of the rat NBT-II cells following exposure to EGF, TGFα, but also to HGF and aFGF (Savagner et al, 1994; Boyer et al, 1989b; Thiery and Chopin, 1999). The fact that only a few cells acquired a mesenchymal phenotype, even after several days of GF treatment, suggests that this transition is an intrinsic property of a very small part of the cellular population. This seems relevant to the in vivo situation where only very few cells achieve metastatic features. The CAL 29 cell line thus offers

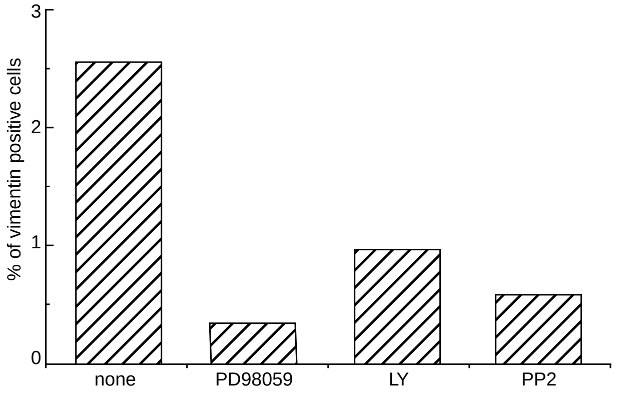

**Figure 7** Effect of different kinase inhibitors on CAL 29 phenotype transition. Prior to addition of TGFα, the cells were preincubated with three different kinase inhibitors: PD 98059 for MEK, LY for PI3-kinase, and PP2 for Src-like kinase. The number of cells expressing vimentin was evaluated by immunofluorescence labelling after three days of culture. Similar results were obtained with EGF (not shown)

the opportunity to dissect the molecular events leading to vimentin expression. The purification of these vimentin-positive cells and the comparative analysis of gene expression by vimentin-positive and negative cells should give crucial information about the emergence of potentially metastatic cells. It is noteworthy that the non-tumorigenic CAL 185 cell line neither scattered nor expressed vimentin intermediary filaments in the presence of GF.

As demonstrated, specific inhibitors of PI-3 kinase, MEK and Src-like kinases strongly impaired the GF-induced cell spreading and vimentin expression. These results suggest that these signalling molecules might be important components of the cellular responses to EGF and TGFα leading to EMT. This approach has been used in the NBT-II model to study the involvement of signalling molecules such as Ras, Src, PKC and CRK in the adhesion and motility of these epithelial cells (Boyer et al, 1997; Petit et al, 1999; Petit et al, 2000). These pharmacological agents could help to understand the intracellular pathways involved in invasiveness and to find new targets for limiting metastasis.

Finally, in preliminary experiments, we have been able to culture urine sediments from patients with superficial urothelial carcinomas and to analyse TGFα-induced EMT in vitro. We have shown in several cases, that these cells acquired a mesenchymal phenotype characterised by intermediary filaments type vimentin (data not shown) but further analysis are necessary to correlate these data with the clinical evolution of these patients.

### ACKNOWLEDGEMENTS

The authors thank Ms A Doye for excellent technical assistance and Ms A Grima and Mr C Minghelli (INSERM ADR C2) for artwork.

### Grant sponsor

Nancy Cattan is a BIOMED fellow. This work was supported by BIOMED grant BMH4-CT96-1439.

### REFERENCES

Behrens J, Mareel MM, Van Roy FM and Birchmeier W (1989) Dissecting tumor cell invasion: Epithelial cells acquire invasive properties after

 *© 2001 Cancer Research Campaign*

the loss of uvomorulin-mediated cell-cell adhesion. *J Cell Biol* **108**: 2435–2447

Birchmeier C, Birchmeier W and Brand-Saberi B (1996) Epithelial-mesenchymal transitions in cancer progression. *Acta Anat* **156**: 217–226

Boyer B, Tucker GC, Valles AM, Franke WW and Thiery JP (1989a) Rearrangements of desmosomal and cytoskeletal proteins during the transition from epithelial to fibroblastoid organization in cultured rat bladder carcinoma cells. *J Cell Biol* **109**: 1495–1509

Boyer B, Tucker GC, Valles AM, Gavrilovic J and Thiery JP (1989b) Reversible transition towards a fibroblastic phenotype in a rat carcinoma cell line. *Int J Cancer (***Suppl***)* **4**: 69–75

Boyer B, Roche S, Denoyelle M and Thiery JP (1997) Src and Ras are involved in separate pathways in epithelial cell scattering. *EMBO J* **16**: 5904–5913

Cooper CS and See WA (1992) The impact of iatrogenic urothelial trauma on urinary levels of transforming growth factor-alpha. *J Urology* **147**: 1647–1649

Dutrillaux B and Lejeune J (1971) Sur une nouvelle technique d'analyse du caryotype humain. *CR Acad Sci* **272**: 2638–2642

Gioanni J, Zanghellini E, Mazeau C, Michiels JF, Crafa F, Gugenheim J, Loubiere R, Schneider M and Demard F (1993) An immunophenotyping procedure for human carcinomas using a panel of nine new monoclonal antibodies. *Oncol (Life Sci. Adv.)* **12**: 89–100

Gilles C, Polette M, Piette J, Delvigne AC, Thompson EW, Foidart JM and Birembaut P (1996) Vimentin expression in cervical carcinomas: association with invasive and migratory potential. *J Pathol* **180**: 175–180

Gravilovic J, Moens G, Thiery JP and Jouanneau J (1990) Expression of transfected transforming growth factor alpha induces a motile fibroblast-like phenotype with extracellular matrix-degrading potential in a rat bladder carcinoma cell line. *Cell Regul* **1**: 1003–1014

Hay ED (1995) An overview of epithelio-mesenchymal transformation. *Acta Anat* **154**: 8–20

Imai T, Kimura M, Takeda M and Tomita Y (1995) Significance of epidermal growth factor receptor and c-erbB-2 protein expression in transitional cell cancer of the upper urinary tract for tumor recurrence at the urinary bladder. *Br J Cancer* **71**: 69–72

Khwaja A, Lehmann K, Marte BM and Downward J (1998) Phosphoinositide-3-kinase induces scattering and tubulogenesis in epithelial cells through a novel pathway. *J Biol Chem* **273**: 18793–18801

Lochter A, Galosy S, Muschler J, Freedman N, Werb Z and Bissell MJ (1997) Matrix metalloproteinase stromelysin-1 triggers a cascade of molecular alterations that leads to stable epithelial-to-mesenchymal conversion and a premalignant phenotype in mammary epithelial cells. *J Cell Biol* **139**: 1861–1872

Mellon J, Cook S, Chambers P and Neal D (1996) Transforming growth factor alpha and epidermal growth factor levels in bladder cancer and their relationship to epidermal growth factor receptor. *Br J Cancer* **73**: 654–658

Petit V, Boyer B, Thiery JP and Valles AM (1999) Characterization of the signaling pathways regulating alpha2beta1 integrin-mediated events by a pharmacological approach. *Cell Adhes Commun* **7**: 151–165

Petit V, Boyer B, Lentz D, Turner CE, Thiery JP and Valles AM (2000) Phosphorylation of tyrosine residues 31 and 118 on paxillin regulates cell migration through an association with CRK in NBT-II cells. *J Cell Biol* **148**: 957–970

Ravery V, Colombel M, Popov Z, Bastuji S, Patard JJ, Bel J, Abbou CC, Fradet Y and Chopin DK (1995) Prognosic value of epidermal growth factor-receptor, T138 and T43 expression in bladder cancer. *Br J Cancer* **71**: 196–200

Ruck A and Paulie S (1998) EGF, TGF alpha, AR and HB-EGF are autocrine growth factors for human bladder carcinoma cell lines. *Anticancer Res* **18**: 1447–1452

Savagner P, Valles AM, Jouanneau J, Yamada KM and Thiery JP (1994) Alternative splicing in fibroblast growth factor receptor 2 is associated with induced epithelial-mesenchymal transition in rat bladder carcinoma cells. *Mol Biol Cell* **5**: 851–862

Thiery JP and Chopin D (1999) Epithelial cell plasticity in development and tumor progression. *Cancer Metastasis Rev* **18**: 31–42

*© 2001 Cancer Research Campaign*
